# Effect of ion implantation energy for the synthesis of Ge nanocrystals in SiN films with HfO_2_/SiO_2 _stack tunnel dielectrics for memory application

**DOI:** 10.1186/1556-276X-6-177

**Published:** 2011-02-28

**Authors:** Bhabani Shankar Sahu, Florence Gloux, Abdelilah Slaoui, Marzia Carrada, Dominique Muller, Jesse Groenen, Caroline Bonafos, Sandrine Lhostis

**Affiliations:** 1 InESS, UDS-CNRS, 23 rue du Loess, 67037 Strasbourg, France; 2 Groupe Nanomat, CEMES-CNRS, Université de Toulouse, 29 rue J. Marvig, B.P. 94347, 31055 Toulouse, France; 3 ST Microelectronics, 850 rue Jean Monnet, 38926 Crolles, France

## Abstract

Ge nanocrystals (Ge-NCs) embedded in SiN dielectrics with HfO_2_/SiO_2 _stack tunnel dielectrics were synthesized by utilizing low-energy (≤5 keV) ion implantation method followed by conventional thermal annealing at 800°C, the key variable being Ge^+ ^ion implantation energy. Two different energies (3 and 5 keV) have been chosen for the evolution of Ge-NCs, which have been found to possess significant changes in structural and chemical properties of the Ge^+^-implanted dielectric films, and well reflected in the charge storage properties of the Al/SiN/Ge-NC + SiN/HfO_2_/SiO_2_/Si metal-insulator-semiconductor (MIS) memory structures. No Ge-NC was detected with a lower implantation energy of 3 keV at a dose of 1.5 × 10^16 ^cm^-2^, whereas a well-defined 2D-array of nearly spherical and well-separated Ge-NCs within the SiN matrix was observed for the higher-energy-implanted (5 keV) sample for the same implanted dose. The MIS memory structures implanted with 5 keV exhibits better charge storage and retention characteristics compared to the low-energy-implanted sample, indicating that the charge storage is predominantly in Ge-NCs in the memory capacitor. A significant memory window of 3.95 V has been observed under the low operating voltage of ± 6 V with good retention properties, indicating the feasibility of these stack structures for low operating voltage, non-volatile memory devices.

## Introduction

During the last decade, non-volatile memory (NVM) structures consisting of semiconductor nanocrystals (NCs), in particular, Si and Ge-NCs, embedded in a dielectric matrix have drawn considerable attraction because of their high endurance, low operating voltage, reduced lateral discharge path, low power consumption, larger retention, and faster operation [1-5]. Compared to Si-NC, utilization of Ge-NC as the floating gate material can give rise to enhanced device performance because of its smaller band gap, which provides both a higher confinement barrier for retention mode and a lower barrier for program/erase mode [4,5]. Quantum confinement effects should also be higher in Ge than in Si because of its smaller electron and hole effective masses, higher dielectric constant, and larger excitonic Bohr radius [6,7]. In recent studies, high-*k *gate dielectrics replaced the conventional SiO_2 _dielectric to be used as tunnel and control oxides in NVMs, which allows for a thinner equivalent oxide thickness without sacrificing the non-volatility [8-12]. Furthermore, the thicker physical thickness of the high-*k *dielectrics ensures good retention characteristics, while due to unique band asymmetry with Si, their lower electron barrier height allows for a larger tunneling current at low control gate voltage when the device operates in the programming regime [10,12]. However, the trade-off between program/erase efficiency and data retention remains an important issue. One of the promising ways to improve the trade-off is to use an asymmetric tunnel barrier, which typically consists of double-stack insulating layers having different band-gap energies [13-15]. In previous studies, Wang and Lu [16] have implemented stacked HfO_2_/SiO_2 _tunnel layers and successfully fabricated uniform Ge-NCS with improved charge storage effect using electron-beam evaporation method. However, they have employed relatively thicker dielectric films for the evolution of Ge-NCs. In the present investigation, low-energy ion implantation method, which is fully compatible with the mainstream CMOS technology, has been employed for the formation of Ge-NCs in SiN matrix with thinner HfO_2_/SiO_2 _stack tunnel layers. In addition, taking advantage of the excellent diffusion barrier properties of Si_3_N_4 _[17], well-defined Ge-NCs are expected to be formed in the top nitride layer without any significant diffusion of Ge toward Si/tunnel oxide interface and/or to the surface of control layer by suitably varying the implantation parameters and annealing condition. The dependence of implantation energy for the formation and evolution of Ge-NCs in these stack structures were studied further.

## Experimental details

Before ion implantation, 1.2 nm of SiO_2 _was thermally grown on *p*-type Si (100) substrates (resistivity 1-10 Ω cm). Subsequently, 4.7 nm of HfO_2 _were deposited by metal organic chemical vapor deposition technique. The top SiN layer with a thickness of about 12 nm was then deposited with electron cyclotron resonance plasma-enhanced chemical vapor deposition method under a flow of SiH_4 _and N_2 _(instead of NH_3_) to minimize the H content in the films. Ion implantation in these stack layers were carried out with ^74^Ge^+ ^ions using GeH_4 _gas source for the extraction of Ge. The Ge^+ ^ion implantation was carried out at two different energies of 3 and 5 keV, while the dose was kept constant at 1.5 × 10^16 ^cm^-2^. These two sets of samples implanted at 3 and 5 keV are denoted as A3 and A5, respectively. The post-implanted samples were subjected to conventional furnace annealing at 800°C in highly pure dry N_2 _for 30 min for the evolution of Ge-NCs. For reference, some SiN/HfO_2_/SiO_2 _stack layers were treated under the same annealing condition without any Ge^+ ^implantation and were defined as the control sample. The formation and evolution of Ge-NCs have been investigated using high-resolution electron microscopy (HREM) on cross-sectional specimens. Cross sectional samples were prepared by mechanical polishing and ion milling using the standard procedure. HREM images were taken using a field emission TEM (FEI Tecnai™ F20 operating at 200 kV) equipped with a spherical aberration corrector. Metal-insulator-semiconductor (MIS) memory capacitor structures were fabricated from the samples by evaporating Al electrodes with 0.8-mm diameter with a shadow mask and Al rear-side contact after scratching the back surface. Capacitance-voltage (*C*-*V*) and conductance-voltage (*G*-*V*) measurements were carried out using HP4192A impdance analyzer through a LABVIEW interface.

## Results and discussion

**Figure 1:**
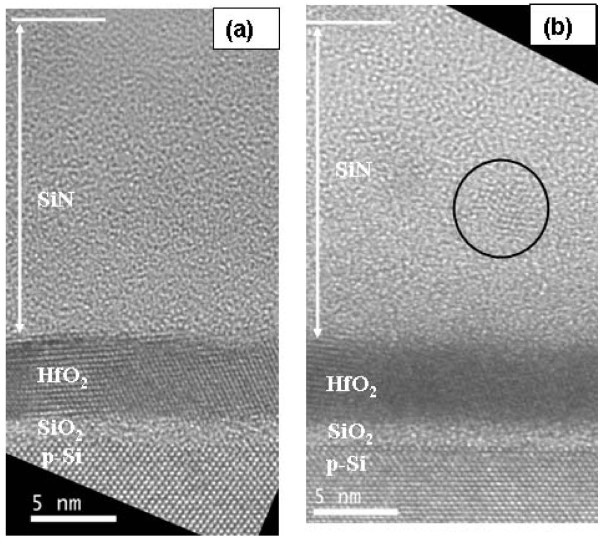
**Cross-sectional HREM images of Ge^+^-implanted SiN/HfO_2_/SiO_2 _stack layers**. Cross-sectional HREM images of Ge^+^-implanted SiN layers with HfO_2_/SiO_2 _stack tunnel dielectrics at two different energies **(a) **3 keV, and **(b) **5 kev, with dose of 1.5 × 10^16 ^cm^-2^, followed by a post-implantation thermal annealing at 800°C in N_2_. In the images, the surface of SiN layer is indicated by a white line

**Figure 2:**
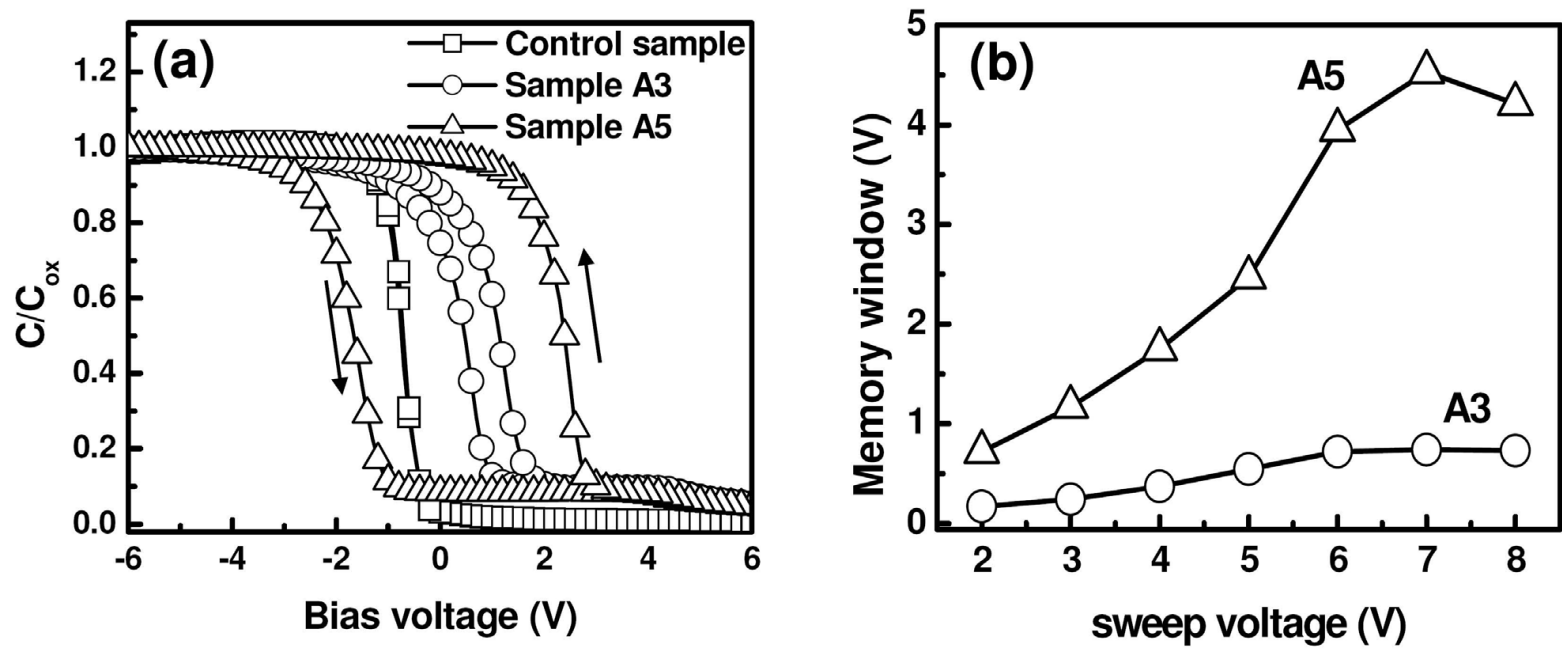
**C-V characteristics of Ge**^**+**^**-implanted and subsequently annealed SiN/HfO_2_/SiO_2 _stack layers**. **(a) **High-frequency (500 kHz) *C*-*V *characteristics of Al/SiN/HfO_2_/SiO_2_/Si MIS structures with Ge-NCs embedded in the SiN layer with HfO_2_/SiO_2 _stack tunnel dielectrics stack layer implanted at two different energies of 3 and 5 keV, along with the control sample, **(b) **variation of memory window (calculated from flat-band shifts) as a function of absolute sweep voltage

Cross-sectional HREM images of the post-implanted annealed samples A3 and A5 are shown in Figure 1a,b, respectively. As evident from Figure 1a, no Ge-NC was observed for sample A3. The SiN layer underwent a swelling of about 4 nm, whereas the thickness of the underlying HfO_2 _and SiO_2 _layers remain almost the same as in the as-deposited sample. In contrast, HREM image of sample A5 (Figure 2b) shows the existence of a Ge-NC with clear lattice fringes with a separation of 0.327 nm, which matches well with the Ge (111) inter planar distance in the diamond structure. Nearly spherical-shaped Ge-NCs with an average size of about 3.5 nm were clearly observed in the SiN matrix at a distance of about 5.6 nm from SiN/HfO_2 _interface. The total SiN thickness (with embedded Ge-NCs) is 15.7 nm, indicating significant swelling of this layer (3.7 nm) as a result of ion implantation and annealing. There is no significant increase of the HfO_2 _thickness while the interfacial SiO_2 _(IL) layer increases from 1.2 to 1.9 nm as a result of implantation and annealing. This swelling could be attributed to the Si substrate oxidation. This phenomenon has already been observed for ion-implanted thin layers and has been attributed to penetration of H_2_O from the ambient through the highly damaged layers [18]. It is noteworthy that the total SiN thickness of both samples after post-implantation thermal annealing is comparable, indicating the weak dependence of swelling effect on implantation energy [19]. As discussed before, for low implantation energies, swelling effect is predominantly dependent on implantation dose rather than implantation energy [19,20].

Figure 2a shows the typical high-frequency (500 kHz) *C*-*V *curves of samples A3, A5, and the control sample. The control sample without any Ge-NC shows a typical high-frequency *C*-*V *curve with negligible hysteresis (0.08 V). The extremely low hysteresis, along with a sharp transition from accumulation and depletion demonstrates the high quality of interfacial as well as bulk properties of these stack layers. In contrast, significant counter-clockwise hysteresis loops are present in the post-implanted annealed samples (A3 and A5), indicating charge trapping in the capacitors. The counter-clockwise nature of *C*-*V *curves is generally attributed to charge storage through substrate injection mechanism [21]. When a positive bias voltage is applied, electrons are being injected from the inversion layer of the Si substrate into the gate dielectric matrix. When a negative voltage is applied, electrons are ejected back into the Si substrate (equivalent to hole injection from the deep accumulation layer of the substrate), resulting in a shift of the *C*-*V *curve toward negative voltages [21,22]. In general, the hysteresis phenomena observed for NCs-embedded MOS structures may be introduced by the mixed effect of injected charges stored in the NC-related traps (traps inside NCs and traps at NC/dielectric interface), essential trap charges existing in the dielectric matrix, or the interface states between the dielectric and Si substrate [8]. When the interface states dominate, the shape of the *C*-*V *curves will be smeared out. In this study, no smearing-out effect was observed for the memory structures with or without Ge-NCs, and the *C*-*V *curves show a sharp transition from accumulation to inversion, indicating a low density of interface states in the samples of this study. Indeed, Ge-NCs are expected to act as predominant charge traps and to produce significant hysteresis, as previously reported [23]. This is consistent with the fact that the largest hysteresis loop is observed for sample A5, which has clearly defined Ge-NCs, while reduced hysteresis has been observed for sample A3, where no obvious Ge-NC was observed. However, the observed memory window for sample A3 can be attributed to charge trapping in Ge-related defect states within the SiN dielectrics. In addition, the lateral charge loss in this sample cannot be ignored because of the absence of discrete charge storage nodes, which generally gives rise to reduced charge storage with a smaller memory window. Figure 2b exhibits the variation of hysteresis memory window due to increasing the sweep voltage from ± 2 to ± 8 V. A maximum memory window of 0.74 and 4.53 V are obtained at a sweep voltage of ± 7 V for sample A3 and A5, respectively. The magnitude of trapped charge density can be estimated using the relation [24]

$$N_{\text{charge}}=\Delta V_{\text{fb}}\times C_{\text{ox}}/qA,$$

where Δ*V*_fb _is the measured flat-band shift, *C*_ox _is the total oxide capacitance, *q *is the electronic charge, and *A *is the top contact area. The trap charge density was estimated to be 5.7 × 10^12 ^cm^-2 ^(sample A5) and 0.78 × 10^12 ^cm^-2 ^(sample A3) at a sweeping voltage of ± 7 V, indicating that the significant charge storage in sample A5 is predominantly due to Ge-NCs. It is interesting to note that the *C*-*V *curve of sample A3 shows a significant positive shift compared to the control sample, indicating the existence of fixed negative charges in the dielectrics. It is speculated that sample A3 contains a significant amount of GeO_*x*_-type network. These dangling bond structures can then capture electrons and become negatively charged, thereby causing a positive shift of the *C*-*V *curves of sample A3. Similar observations have been reported for Ge-NCs embedded in a SiO_2 _matrix [23].

**Figure 3:**
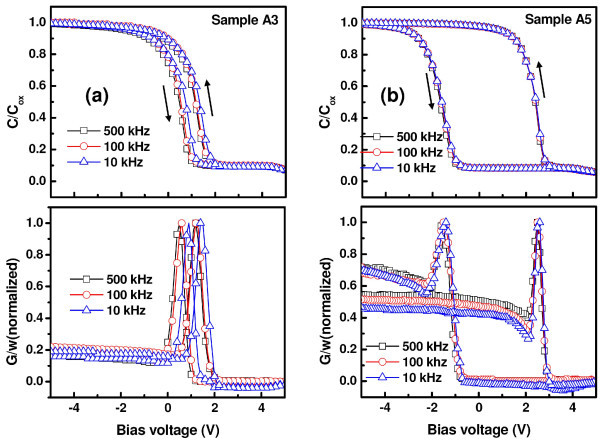
**(Color online) Frequency dependent C-V and G-V characteristics**. Frequency-dependent *C*-*V *and *G*-*V *characteristics of Al/SiN/HfO_2_/SiO_2_/Si MIS structures with Ge^+ ^implanted at two different energies **(a) **3 keV (sample A3) and **(b) **5 keV (sample A5) taken in the frequency range of 10-500 kHz

**Figure 4:**
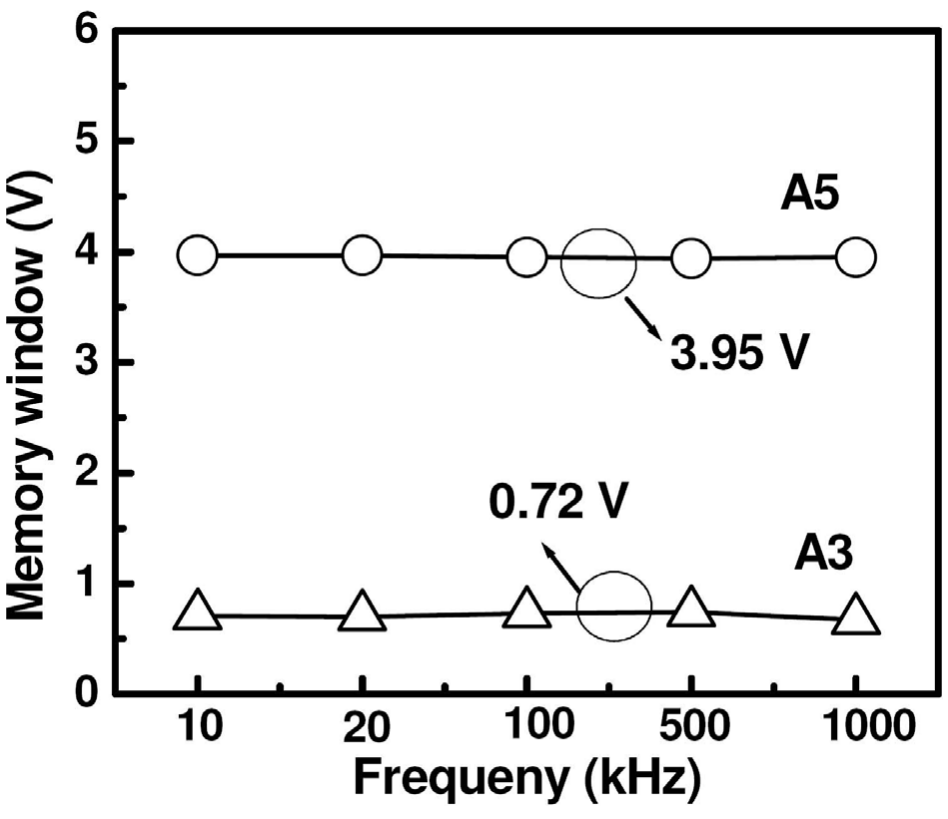
**Frequency dependent C-V hysteresis windows**. **The *C*-*V *hysteresis windows measured at various frequencies ranging from 10 kHz to 1 MHz for the two samples at a sweep voltage of ± 6 V**. The observed memory windows maintain the same value of 3.95 and 0.72 V for the applied frequencies in samples A5 and A3, respectively.

For a better understanding of the results, frequency-dependent *C*-*V *and *G*-*V *measurements were further carried out in the frequency range of 10-500 kHz. This is to ascertain that most of the charging effect originated mainly from Ge-NCs and/or Ge-NCs-related traps with minimal influence from interface traps, which typically lead to frequency dispersion in *C*-*V *and *G*-*V *characteristics. For this purpose, *G*-*V *measurement is considered to be a more sensitive approach than *C*-*V *measurement technique and provides the dynamic information related to trap density [25-27]. In fact, conductance is directly related to the energy loss in response to the applied AC signal during the capture and emission of charge carriers by interface states. Frequency-dependent *C*-*V *and *G*/*w*-*V *curves for sample A3 and A5 are shown in Figure 3a,b, respectively. In both cases, no distortion in *C*-*V *characteristics due to slow traps and/or large surface density (flat step) was observed in the samples under study with a change in frequency. It was noticed that the full-width-at-half-maximum (FWHM) of the conductance peak is small and almost constant in the frequency range of 10-500 kHz, indicating that the hysteresis and conductance peak are of the same origin [28]. It is a well-known fact that a conductance peak with large FWHM values can be attributed to the presence of a considerable amount of interface states. It is interesting to note that both *C*-*V *and *G*/*w*-*V *curves of sample A3 shift toward more positive bias with decreasing frequency, and the shift is more prominent in the low-frequency region (<50 kHz). The shift is marked by minimal frequency dispersion in accumulation (less than 2%), capacitance indicating minimal influence of series resistance, and dielectric constant variation with altering the measurement frequency. From Figure 4, it is noteworthy that the same amount of hysteresis and stored charge were obtained in sample A3 irrespective of the measurement frequency. Hence, the capacitance shift can be attributed to the presence of fast traps and/or border traps (near-interfacial traps), which can have a rapid communication with the underlying Si-substrate [29]. The *G*/*w*-*V *curves shift in accordance with the *C*-*V *curves. It was observed that *G*/*w*-*V *curves of sample A3 exhibit broader and larger peaks near flat-band voltage than those of sample A5, indicating higher energy loss during charge exchange.

**Figure 5:**
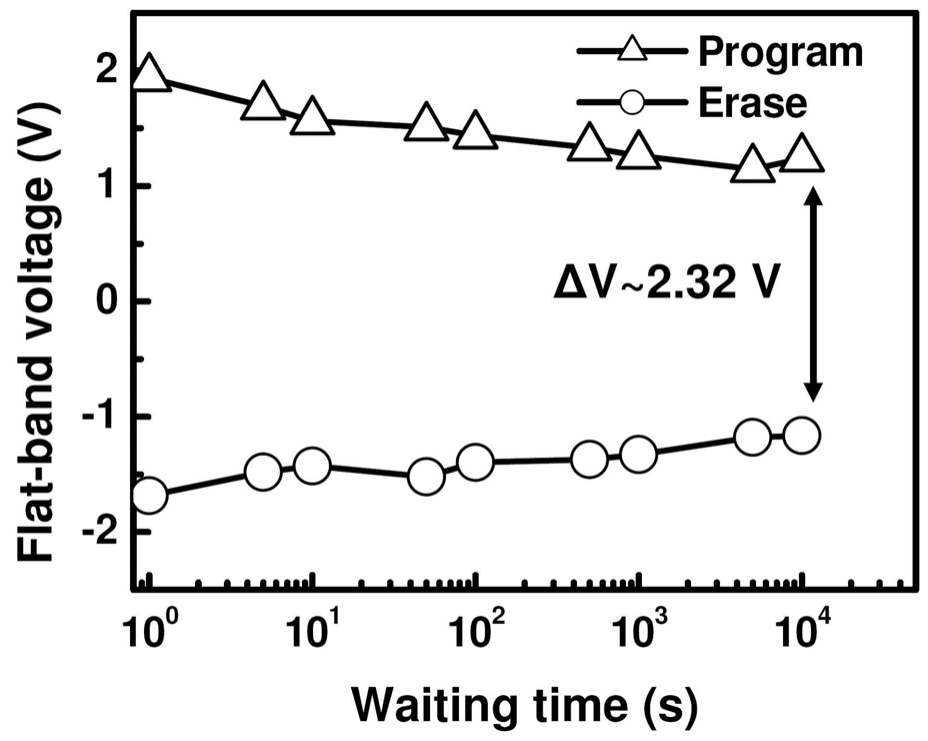
**Evolution of flat-band voltage as a function of waiting time for the MIS structures after Ge^+ ^ion implantation at 5 keV followed by thermal annealing at 800°C and subjected to a stress voltages of +6 V for electron charging, and -6 V for hole charging for 3s**. The constant-capacitance method at flat-band point was used for this measurement.

In the investigation of this study, an implantation energy of 5 keV seems to be the optimum parameter for a particular dose of 1.5 × 10^16 ^cm^-2 ^and SiN/HfO_2_/SiO_2 _stack for the evolution of Ge-NCs and obtaining significant memory properties. In this regard, sample A5 has been chosen for charge retention measurement to have a better insight for its utility in low power-consuming NVM devices. Figure 5 shows the charge retention characteristics of sample A5 by stressing the samples with voltage pulses of ± 6 V (positive for electron charging and negative for hole charging) for 3s. The retention curves exhibit a logarithmic dependence on the waiting time. A faster charge loss rate was observed after applying a positive stress, indicating higher electron loss rate due to the higher conductance band edge of Ge-NCs [30]. A significant memory window of 2.36 V has been achieved through a waiting time of 10^4 ^s with a possible trend of stabilization indicating charge confinement in Ge-NCs. With further extrapolation of the retention curves, a memory window of 1.06 V has been estimated after a waiting time of 10 years. This enhanced charge retention should be attributed to charge confinement in Ge-NCs, immunity of Ge-NCs to local defects in the dielectric, and interface traps.

## Conclusions

In summary, we have conducted a comparative investigation of Ge^+ ^ion implantation energy-dependent memory effects in SiN dielectric layers with HfO_2_/SiO_2 _asymmetric tunnel barriers at a constant implantation dose of 1.5 × 10^16 ^cm^-2^, and subsequent thermal annealing at 800°C in N_2_. For the lower Ge^+ ^implantation energy of 3 keV, no Ge-NC was observed in the stack structures, and the resultant MIS structure exhibited a small memory window of 0.74 V, which is attributed to a net negative charge storage in GeO_*x*_-dangling bonds. In contrast, for the higher Ge^+ ^implantation energy of 5 keV, nearly spherical and well-isolated Ge-NCs with an average size of 3.5 nm were self-assembled within the top Si_3_N_4 _layer at a distance of 5.6 nm from SiN/HfO_2 _interface. A significant memory window of 3.95 V has been achieved over a small voltage sweep range (≤6 V). Frequency-dependent *C*-*V *and *G*-*V *curves indicate negligible contribution from interfacial defects toward the charge storage capability. An extrapolated memory window of about 1.06 V is achievable for a waiting time of 10 years due to the charge confinement in Ge-NCs, indicating the utility of these Al/SiN/Ge-NC + SiN/HfO_2_/SiO_2_/Si stack structures for low operating voltage NVM devices.

## Abbreviations

FWHM: full width at half maximum; HREM: high-resolution electron microscopy; MIS: metal-insulator-semiconductor; NCs: nanocrystals; NVM: non-volatile memory.

## Competing interests

The authors declare that they have no competing interests.

## Authors' contributions

BSS, AS, MC, and CB designed the study. SL provides the HfO_2_/SiO_2 _layer. Then BSS developed the SiN/HfO_2_/SiO_2 _stack layers. DM helped in ion implantation in these stack structures. FG, MC, JG, and CB prepared the samples for TEM observation and investigated the TEM results and done all the TEM analysis. BSS investigated and performed post-fabrication treatment, carried out all the electrical characterization studies, analyzed the results, and prepared the draft of the manuscript. Moreover, AS and CB participated in the coordination of study. All authors read and approved the final manuscript.
